# Posterior Reversible Encephalopathy Syndrome (PRES) as the Initial Presentation of Systemic Lupus Erythematosus With Lupus Nephritis: A Case Report

**DOI:** 10.7759/cureus.107244

**Published:** 2026-04-17

**Authors:** Cheyenne De Clercq, Amédée Ego, Sarah Heenen, Dominique Vandervelde

**Affiliations:** 1 Emergency Department, HUmani - CHU (Centre Hospitalier Universitaire) Charleroi-Chimay, Charleroi, BEL; 2 Intensive Care Unit, HUmani - CHU (Centre Hospitalier Universitaire) Charleroi-Chimay, Charleroi, BEL; 3 Intensive Care Unit, Hopitaux Iris Sud, Brussels, BEL; 4 Nephrology Department, Hopitaux Iris Sud, Brussels, BEL

**Keywords:** glomerulonephritis, hypertensive emergency, lupus nephritis, nephrology, posterior reversible encephalopathy syndrome (pres), seizures, systemic lupus erythematosus (sle)

## Abstract

Posterior reversible encephalopathy syndrome (PRES) is a clinico-radiological entity most often associated with acute hypertension, renal failure, or autoimmune diseases. PRES as an initial manifestation of systemic lupus erythematosus (SLE) has been described but remains rare. Early recognition and treatment of the underlying condition are essential to ensure a favorable outcome.

We report the case of an 18-year-old North African female residing in Belgium, admitted for gastrointestinal symptoms associated with severe arterial hypertension, acute renal failure, and neurological manifestations, including seizures and visual disturbances.

Brain magnetic resonance imaging (MRI) showed bilateral posterior and basal nuclei hyperintensities consistent with PRES. Laboratory investigations revealed impaired renal function and immunological abnormalities, including positive antinuclear and anti-double-stranded DNA antibodies. Further evaluation confirmed lupus nephritis, establishing the diagnosis of SLE.

Treatment included prompt blood pressure control, antiepileptic therapy, high-dose corticosteroids, and immunosuppressive therapy targeting lupus nephritis.

The patient experienced complete neurological recovery with resolution of seizures and visual symptoms. Follow-up imaging demonstrated radiological improvement, and renal function gradually recovered under immunosuppressive treatment.

This case illustrates PRES as a rare inaugural manifestation of SLE in the context of hypertensive emergency and lupus nephritis. Early diagnosis and management of both neurological and systemic involvement are crucial to achieve complete neurological recovery and improve long-term outcomes.

Strict blood pressure control and timely evaluation for SLE are critical in suspected PRES, and clinicians should be aware that atypical involvement of the basal ganglia may occur.

## Introduction

Posterior reversible encephalopathy syndrome (PRES) is a rare clinico-radiological entity characterized by acute neurological symptoms, including seizures, headaches, visual disturbances, and altered mental status, typically triggered by acute hypertension, renal failure, or autoimmune diseases [1]. It is thought to result from impaired cerebral autoregulation and endothelial dysfunction. Although the exact incidence remains uncertain, PRES is considered an uncommon condition and is likely underdiagnosed due to variable clinical presentations. 

Systemic lupus erythematosus (SLE) is a multisystem autoimmune disease that may involve renal and neurological complications. PRES has been increasingly reported in patients with SLE, particularly in association with lupus nephritis, severe hypertension, and active disease. However, PRES as the initial manifestation of previously undiagnosed SLE remains uncommon, with reported prevalence ranging from approximately 0.4% to 0.7% in SLE cohorts [2,3]. Early recognition and management of both PRES and its underlying causes are crucial for achieving favorable outcomes [1].

This case report discusses an 18-year-old female with PRES as the inaugural manifestation of SLE, emphasizing the importance of timely intervention, prompt blood pressure control, early autoimmune evaluation, and awareness of atypical radiological patterns.

## Case presentation

An 18-year-old North African patient living in Belgium was admitted to the emergency room. She reported having abdominal pain, cramps, nausea, vomiting, and diarrhea for four days. She reported chills without documented fever. She also described asthenia, headaches, slight photophobia, and blurred vision for the last few days. She had no urinary complaints and had not traveled recently. She had no known past medical history. However, during her last visit to her general practitioner, he noted a new-onset arterial hypertension and bilateral lower extremity edema. He also noticed significant weight gain and mild hypercholesterolemia on her blood test. The patient was a high school student with a sedentary lifestyle and frequent intake of processed foods. She did not consume alcohol or use tobacco.

On admission to the emergency room, her blood pressure was 180/128 mmHg. She was tachycardic at 127 beats per minute. She was afebrile and had a good oxygen saturation. On physical examination, she was pale and tired. Her cardiopulmonary examination was normal. Her abdomen was slightly tender. She had lower limb edema. The neurological examination was normal, with no neck stiffness and no neurological deficits.

Laboratory findings at admission are summarized in Table 1. Blood tests revealed hypochromic normocytic anemia, a mild inflammatory syndrome, and severe acute renal failure (creatinine 5.96 mg/dL, estimated glomerular filtration rate (eGFR) 10 mL/min/1.73 m²) with electrolyte disturbances including hyperphosphatemia, hyperkalemia, and metabolic acidosis. Hypoalbuminemia and marked hypercholesterolemia were also noted. Beta-hCG, HIV serology, and toxicology screening were negative, helping exclude pregnancy-related hypertensive disorders, infectious causes, and toxic etiologies. Electrocardiogram and echocardiography were normal.

**Table 1:** Laboratory findings at admission CRP: C-reactive protein; eGFR: estimated glomerular filtration rate; CKD-EPI: Chronic Kidney Disease Epidemiology Collaborative Group; β-hCG: beta-human chorionic gonadotropin; HIV: human immunodeficiency virus

Parameter	Result	Unit	Reference Range
Hemoglobin	9.9	g/dL	12.0–15.0
White Blood Cells	11.17	×10³/µL	3.9–9.0
Neutrophils (absolute)	8.61	×10³/µL	
CRP	23.06	mg/L	<5.0
Urea	80.0	mg/dL	18.0–45.0
Creatinine	5.96	mg/dL	0.50–0.90
eGFR (CKD-EPI)	10	mL/min/1.73m²	>60
Phosphate	3.35	mmol/L	0.80–1.44
Potassium	5.2	mmol/L	3.5–5.1
Bicarbonate	11	mmol/L	22–29
Glucose	110	mg/dL	70–100
Albumin	27	g/L	32–45
Total Cholesterol	304	mg/dL	<190
HDL Cholesterol	45.3	mg/dL	>40
LDL Cholesterol	220	mg/dL	<200
Triglycerides	191	mg/dL	<150
β-hCG	Negative	—	Negative
HIV Serology	Negative	—	Negative

In the emergency room, she developed a tonic-clonic seizure that resolved spontaneously after one minute. She presented an altered level of consciousness postictal with episodes of severe agitation. A cerebral CT with contrast did not reveal intracerebral hemorrhage or vascular abnormality. A lumbar puncture showed crystal-clear cerebrospinal fluid (CSF) with normal opening pressure. CSF analysis is summarized in Table 2. It revealed five nucleated cells, hypoglycorrhachia, normal protein, and normal lactate. The molecular biology multiplex panel was negative. These findings made infectious meningitis or encephalitis unlikely.

**Table 2:** Cerebrospinal fluid analysis at admission *The glucose reference range depends on serum glucose.

Parameter	Result	Unit	Reference Range
Opening pressure	Normal	cm H₂O	10–20
Nucleated cells	5	/µL	0–5
Glucose	46	mg/dL	50–80*
Protein	0.264	g/L	0.15–0.45
Lactate	1.7	mmol/L	1.1–2.4
Multiplex polymerase chain reaction panel	Negative	—	Negative

Given the combination of hypertensive emergency, acute renal failure, and neurological symptoms, initial differential diagnoses included hypertensive encephalopathy, intracranial infection, cerebral venous thrombosis, metabolic encephalopathy, and autoimmune disease flare.

**Figure 1:**
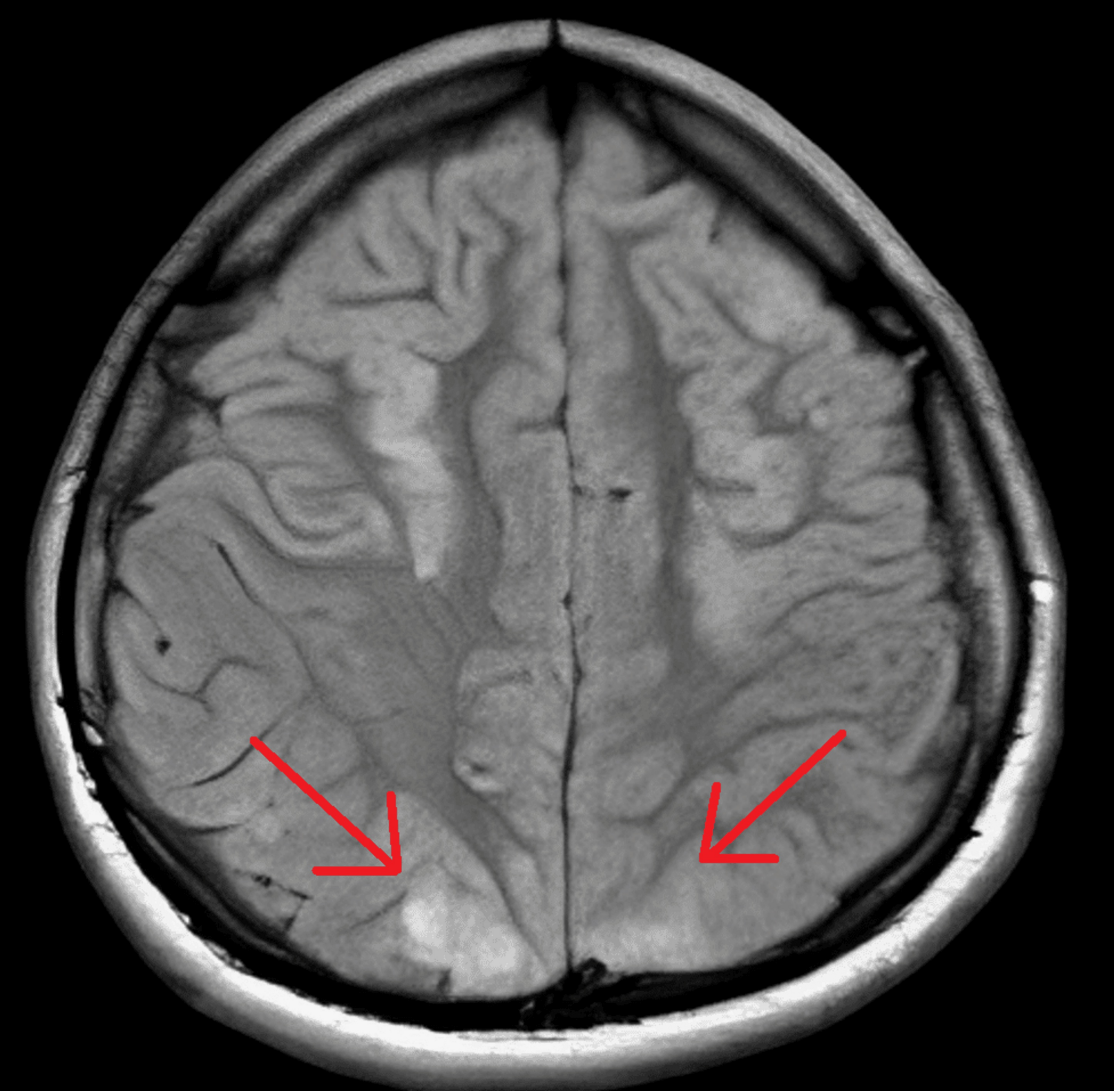
Cranial magnetic resonance imaging (FLAIR sequence) Bilateral posterior symmetric hyperintensities consistent with vasogenic edema (red arrows) FLAIR: fluid-attenuated inversion recovery

She was admitted to the intensive care unit (ICU) for agitation, and dexmedetomidine was initiated. Blood pressure was treated with continuous intravenous nicardipine infusion, allowing gradual reduction and stabilization of blood pressure over the following 24-48 hours. Levetiracetam 500 mg twice a day was also started as an antiseizure medication. Brain magnetic resonance imaging (MRI) was performed the same day and showed hyperintensities ​​​ in the occipital lobes and in the basal nuclei with a symmetrical aspect on T2-weighted and fluid-attenuated inversion recovery (FLAIR) sequences, with no evidence of venous sinus thrombosis, ischemia, or hemorrhage (Figure 1). An electroencephalogram demonstrated non-specific encephalopathy without epileptic activity. At the time, the medical team considered PRES of initially unclear etiology.

During the same day, continuous renal replacement therapy (cRRT) was initiated for anuria and metabolic complications of acute renal failure. Urine analysis revealed nephrotic-range proteinuria with a protein-to-creatinine ratio of 12.07 g/g, hypoalbuminemia (27 g/L), hypercholesterolemia, and microscopic hematuria, consistent with nephrotic syndrome (Table 3). An ultrasound of the urinary tract was normal. The following day, the autoimmune panel showed speckled antinuclear antibodies (ANA), strongly positive at a dilution of 1/320, and positive anti-double-stranded DNA (anti-dsDNA) antibodies. Complement levels (C3, C4) and secondary causes of nephrotic syndrome were investigated.

**Table 3:** Urine analysis at admission

Parameter	Result	Unit	Reference Range
Proteinuria	7.87	g/L	<0.15
Protein/Creatinine Ratio	12.07	g/g creatinine	<0.20
Microscopic Hematuria	325	/µL	<20
Urinary Crystals	Present (amorphous crystals)	—	Absent

**Figure 2:**
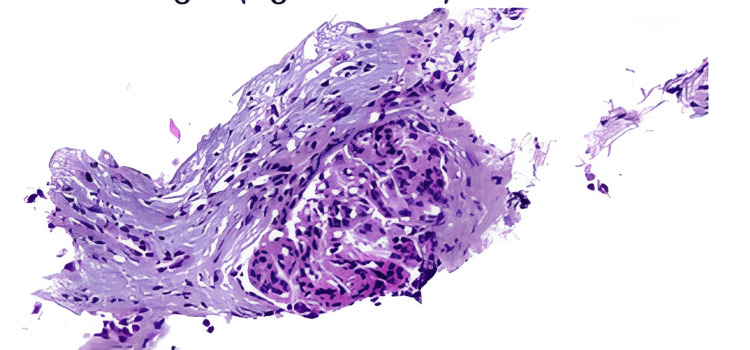
Microscopic image of glomerulonephritis with proliferation of mesangial cells

**Figure 3:**
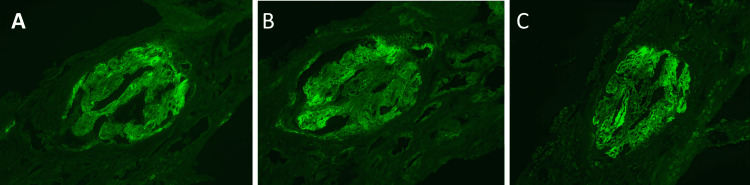
Pathology: immunofluorescence images showing mesangial deposits of IgG, C3, and C1q in glomerulonephritis with mesangial cell proliferation and inflammatory infiltration A: Mesangial deposits of IgG B: Mesangial deposits of complement component C3 C: Mesangial deposits of complement component C1q

Considering an autoimmune etiology, high-dose intravenous corticosteroids (500 mg once daily for three days) followed by oral prednisone 32 mg once daily were initiated after a percutaneous renal biopsy was performed on day 3. Histopathological examination showed a mesangial proliferative glomerulonephritis consistent with lupus nephritis (ISN/RPS Class II), characterized by mesangial immune complex deposition, absence of significant endocapillary proliferation or crescent formation, and minimal chronic changes (Figures 2-3).

Mycophenolate mofetil and hydroxychloroquine were introduced due to severe nephrotic-range proteinuria and acute renal impairment, suggesting possible lupus podocytopathy despite class II histology, in accordance with Kidney Disease: Improving Global Outcomes (KDIGO) recommendations for patients with class I-II lupus nephritis presenting with nephrotic syndrome. Her neurological status gradually improved, the visual disturbances resolved, and her epileptic seizures did not recur. She progressively recovered urine output, allowing transition from continuous renal replacement therapy to intermittent hemodialysis. The intravenous antihypertensive drug was substituted with oral treatment: moxonidine, bisoprolol, amlodipine, and lisinopril. A prophylaxis with trimetoprim-sulfamethoxazole was initiated, as well as erythropoietin. The patient was discharged from the ICU on the tenth day.

A follow-up cerebral MRI performed 11 days later showed a regression of white matter hyperintensities​​​​​. The electroencephalogram was normalized, and she achieved ​​​​​complete neurological recovery. She was discharged home from the hospital on day 20 with a nephrology outpatient clinic follow-up, and long-term immunosuppressive therapy was planned​​​​​. She no longer required intermittent renal replacement therapy (iRRT) on day 30. 

Two months later, renal function had partially recovered, with urea 64 mg/dL and creatinine 1.53 mg/dL (eGFR 49 mL/min/1.73 m²). Proteinuria decreased to 4.2 g/L with albuminuria at 3285 mg/L. Serum albumin and hemoglobin improved to 32 g/L and 10.8 g/dL, respectively.​​​​​​​

Over a two-year follow-up, the patient demonstrated a favorable renal outcome. At one year, renal function was partially recovered (creatinine 1.15 mg/dL, eGFR 70 mL/min/1.73 m²) with low-grade proteinuria (0.71 g/g) and a Systemic Lupus Erythematosus Disease Activity Index (SLEDAI​​​) score of 0. At two years, renal function remained stable with preserved GFR, persistent mild proteinuria, and no clinical signs of nephrotic syndrome, reflecting overall disease stability under ongoing immunosuppressive therapy.

## Discussion

This report describes PRES, an acute or subacute neurotoxic syndrome characterized by vasogenic edema related to the failure of cerebral autoregulation and endothelial dysfunction, occurring in the context of SLE and hypertensive emergency [3,4]. The pathophysiology of PRES is debated and incompletely understood; it seems to be multifactorial, involving endothelial dysfunction with extravasation of plasma proteins into the extracellular space and impaired cerebral autoregulation leading to vasogenic edema [4]. Although acute hypertension is a frequent trigger, PRES also occurs in conditions such as preeclampsia, hypertensive emergency, allogeneic bone marrow and solid organ transplantation, chemotherapy or cytotoxic drugs, and autoimmune diseases, as in our case [1].

PRES is difficult to diagnose because it presents in a variety of ways and occurs infrequently. The diagnosis may be challenging because clinical presentation is heterogeneous and radiological patterns may overlap with encephalitis, toxic-metabolic encephalopathy, demyelinating disorders, or central nervous system vasculitis. The diagnosis is based on a combination of clinical, radiological, and electroencephalographic findings. The clinical presentation is non-specific and may include asthenia, headaches, visual disturbances, which may be hallucinations, decreased acuity, altered consciousness ranging from confusion to encephalopathy, and epileptic phenomena [5]. Nausea and vomiting are also described, and, more rarely, focal neurological deficits [6].

MRI is the cornerstone of diagnosis in T2-weighted and FLAIR sequences. Typical findings include bilateral vasogenic edema predominantly affecting the parieto-occipital white matter. However, atypical involvement is increasingly recognized, including the frontal and temporal lobes, cerebellum, brainstem, and basal ganglia. Hemorrhagic complications, cytotoxic edema, and contrast enhancement have also been described in more severe presentations [3,7,8]. Generally, radiographic abnormalities regress with the resolution of symptoms [9]. The electroencephalogram makes it possible to evaluate the degree of the encephalopathy and to monitor non-convulsant epileptic activity [10].

Since PRES is frequently associated with an altered state of consciousness, patients are regularly admitted to intensive care. Management consists of symptomatic, etiological, and supportive treatments, as well as the control of precipitating causes. First, although there is no recommended blood pressure goal in PRES, it is agreed that in case of hypertensive emergency, blood pressure must not decrease by more than 25% during the first hour of treatment, then gradually normalize over 24-48 hours [11]. Secondarily, antiepileptic drugs should be administered according to the usual recommendations to manage acute seizures, regardless of their underlying cause [12]. This treatment should continue until complete clinical resolution of PRES symptoms. There does not seem to be evidence to prescribe antiepileptic prophylaxis [12].

Finally, the search for a causal factor other than hypertensive emergency should lead to the investigation of autoimmune disease, chemotherapy, or immunomodulatory treatments. It is an idiosyncratic complication and is not associated with administration errors or overdose, and remote reintroduction is not associated with recurrence of PRES [1]. In a case-control study, the prevalence of PRES in patients with SLE has been reported to reach up to 0.43% [2].

SLE is a chronic autoimmune disorder characterized by a wide spectrum of clinical manifestations, from mild cutaneous involvement (malar rash) to severe organ dysfunction, including lupus nephritis. It can impact various organs, including joints, brain, lungs, kidneys, and blood vessels [13]. SLE predominantly affects women, especially those of reproductive age. The first-line treatment is hydroxychloroquine, with glucocorticoids prescribed to manage flare-ups. In cases with a significant risk of organ damage, higher doses of methylprednisolone may be used, and for more severe presentations, long-term immunosuppressive agents are recommended [13].

Lupus nephritis is one of the most common and early complications of SLE, affecting about 50% of patients. It is often identified through early proteinuria. Initial symptoms may include hematuria, increased serum creatinine, lower limb edema, anasarca, and hypertension [13]. Current guidelines recommend hydroxychloroquine for all patients with SLE, with treatment escalation guided by organ involvement and histological class of lupus nephritis. In Class II lupus nephritis, immunosuppressive therapy beyond hydroxychloroquine and renin-angiotensin system blockade is not routinely required. However, the presence of nephrotic-range proteinuria and acute renal impairment may suggest superimposed podocytopathy or evolving disease activity, which may justify additional immunosuppressive therapy on an individualized basis, as reflected in recent KDIGO recommendations [14].

PRES remains an uncommon but recognized neurological complication of SLE, with reported prevalence ranging from approximately 0.4% to 0.7%, depending on cohorts [2,3]. Its presentation is similar to PRES from other causes. Although onset was linked to an SLE flare in more than 90% of cases, other factors, such as hypertension (82-95%), renal insufficiency (73-84%), and immunosuppressive medications (50%), were commonly observed [2]. A study has shown that the SLEDAI score was higher than 6 in patients who developed PRES, indicating higher disease severity at onset [15].

The prompt management of PRES generally leads to good outcomes. Despite its name suggesting reversibility, severe outcomes can occur. In a large hospital-based study, the overall in-hospital mortality rate was 2.2% [16]. In ICU patients with severe PRES, mortality reached 15.7%, with 5.7% directly attributable to PRES [17]. Poor outcomes are associated with neurological complications and delays in diagnosis and treatment [17].

A retrospective study involving 70 ICU patients reported that by day 90, 16% had died, 37% had persistent functional deficits (GOS 2-4), and 56% achieved full recovery (GOS 5) [17].

As in our clinical case, there are reports of lupus glomerulonephritis initially presenting as PRES-type encephalopathy [2,15,18]. In a pooled analysis of 87 cases of PRES associated with lupus erythematosus, the median age was 26.3 years, 85.1% had renal failure, 91.7% had hypertension, and 78.2% had seizures. Five percent had incomplete neurological recovery; risk factors were brain involvement and cerebral hemorrhage (OR 10.9 and 14.0, respectively) [19].

## Conclusions

Posterior reversible encephalopathy syndrome may present with heterogeneous neurological manifestations and can reveal an underlying systemic disease. This case illustrates PRES as the initial manifestation of systemic lupus erythematosus with lupus nephritis in the context of a hypertensive emergency. Diagnosis can be challenging due to variable clinical presentation and overlap with other neurological conditions, requiring a brain MRI for confirmation. Multidisciplinary management is essential to optimize neurological and renal outcomes.

This case underscores the rare association between PRES and SLE, in which PRES may represent the first manifestation of the disease. Clinicians should consider PRES in patients presenting with seizures or encephalopathy in the setting of a hypertensive emergency, and promptly investigate for underlying causes such as autoimmune or renal disease. Early blood pressure control and targeted treatment are key to favorable outcomes.
